# 3,4-Di­amino­pyridinium hydrogen malonate

**DOI:** 10.1107/S1600536813008763

**Published:** 2013-04-10

**Authors:** Surendra Thapa, Sergiu Draguta, Bhupinder Sandhu, Mikhail Yu. Antipin, Tatiana V. Timofeeva

**Affiliations:** a Kathmandu University Budol, Dhulikhel 45200, Nepal; b Department of Chemistry & Biology, New Mexico Highlands University, 803 University Avenue, Las Vegas, NM 87701, USA

## Abstract

In the title salt, C_5_H_8_N_3_^+^·C_3_H_3_O_4_^−^, the 3,4-di­amino­pyridinium cation is almost planar, with an r.m.s. deviation of 0.02 Å. The conformation of the hydrogen malonate anion is stabilized by an intra­molecular O—H⋯O hydrogen bond, which generates an *S*(6) ring. In the crystal, N—H⋯O hydrogen bonds link cations and anions into layers parallel to the *ab* plane.

## Related literature

For applications of 3,4-di­amino­pyridine, see: Maddison *et al.* (2001 ▶); Argov (2009 ▶). For related structures, see: De Cires-Mejias *et al.* (2004 ▶); Koleva *et al.* (2007 ▶, 2008 ▶); Fun & Balasubramani (2009 ▶). For graph-set notation, see: Bernstein *et al.* (1995 ▶).
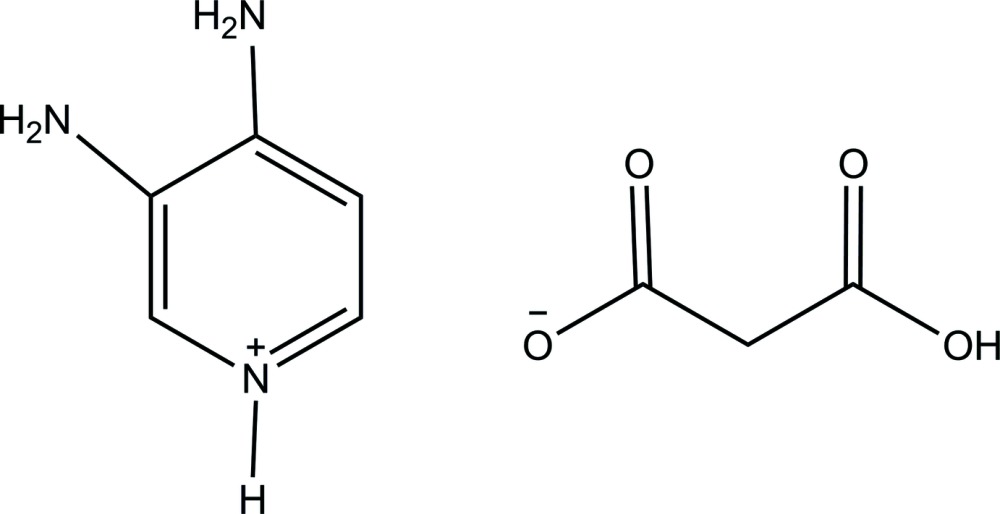


## Experimental

### 

#### Crystal data

C_5_H_8_N_3_^+^·C_3_H_3_O_4_^−^
*M* *_r_* = 213.20
Monoclinic, 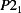

*a* = 8.7761 (18) Å
*b* = 5.088 (1) Å
*c* = 10.636 (2) Å
β = 101.381 (4)°
*V* = 465.58 (17) Å^3^
*Z* = 2
Mo *K*α radiation
μ = 0.12 mm^−1^
*T* = 296 K
0.30 × 0.20 × 0.20 mm

#### Data collection

Bruker APEXII CCD diffractometer
Absorption correction: multi-scan (*SADABS*; Sheldrick, 2003 ▶) *T*_min_ = 0.964, *T*_max_ = 0.976
3498 measured reflections
1248 independent reflections
1066 reflections with *I* > 2σ(*I*)
*R*_int_ = 0.026

#### Refinement

*R*[*F* ^2^ > 2σ(*F* ^2^)] = 0.040
*wR*(*F* ^2^) = 0.102
*S* = 1.18
1248 reflections
140 parameters
1 restraint
H atoms treated by a mixture of independent and constrained refinement
Δρ_max_ = 0.19 e Å^−3^
Δρ_min_ = −0.15 e Å^−3^

### 

Data collection: *APEX2* (Bruker, 2005 ▶); cell refinement: *SAINT* (Bruker, 2001 ▶); data reduction: *SAINT*; program(s) used to solve structure: *SHELXTL* (Sheldrick, 2008 ▶); program(s) used to refine structure: *SHELXTL*; molecular graphics: *SHELXTL*; software used to prepare material for publication: *SHELXTL*.

## Supplementary Material

Crystal structure: contains datablock(s) global, I. DOI: 10.1107/S1600536813008763/cv5392sup1.cif

Structure factors: contains datablock(s) I. DOI: 10.1107/S1600536813008763/cv5392Isup2.hkl

Supplementary material file. DOI: 10.1107/S1600536813008763/cv5392Isup3.cml

Additional supplementary materials:  crystallographic information; 3D view; checkCIF report

## Figures and Tables

**Table 1:** Hydrogen-bond geometry (Å, °)

*D*—H⋯*A*	*D*—H	H⋯*A*	*D*⋯*A*	*D*—H⋯*A*
N1—H1*A*⋯O3	0.86	1.93	2.784 (3)	175
N2—H2*A*⋯O1^i^	0.86	2.28	3.132 (3)	174
N2—H2*B*⋯O1^ii^	0.86	2.15	3.005 (3)	176
N3—H3*A*⋯O2^iii^	0.86	2.28	3.048 (3)	149
N3—H3*B*⋯O1^ii^	0.86	2.10	2.960 (3)	177
O4—H4⋯O2	0.91 (3)	1.55 (3)	2.442 (3)	164 (3)

Symmetry codes: (i) 
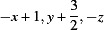
; (ii) 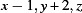; (iii) 
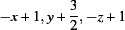
.

**Fig. 1.**
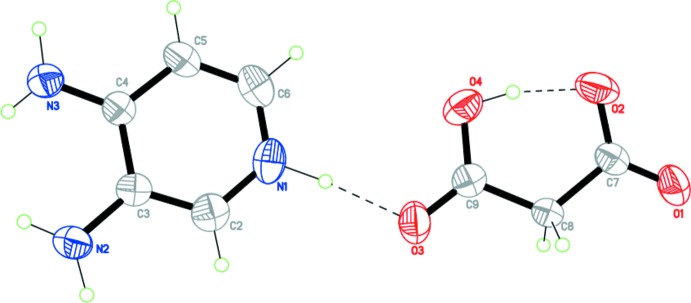
The content of asymmetric unit of I showing the atomic numbering and hydrogen bonds as dashed lines. Displacement ellipsoids are drawn at the 50% probability level. H atoms are presented as small spheres of arbitrary radius.

**Fig. 2.**
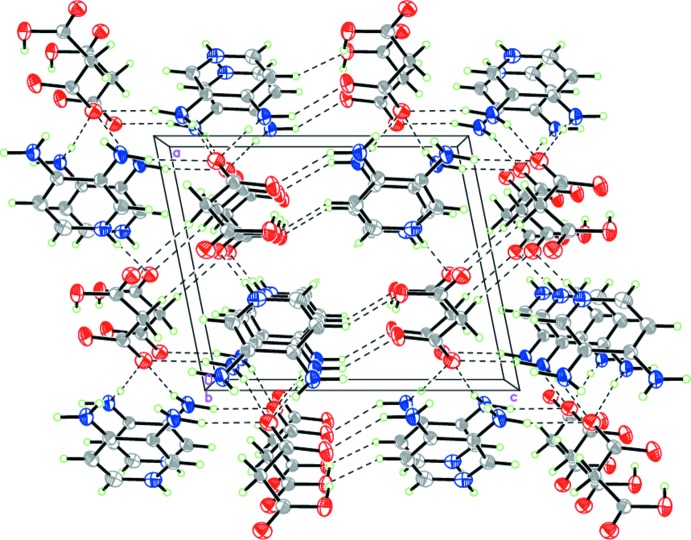
A portion of the crystal packing viewed approximately down the b axis and showing intermolecular N—H···O hydrogen bonds as dashed lines.

## supplementary crystallographic information

### Comment

3,4-Diaminopyridine is used for the treatment of Lambert-Eaton myasthenic syndrome (LEMS) which significantly improve the primary endpoint of muscle strength score, or myometric limb measurement following treatment (Maddison *et al.* 2001). It is also used to treat many of the congenital myasthenic syndromes (Argov, 2009). The crystal structures of adducts of 3,4-diaminopyridine with different acids such as succinic (Fun *et al.*, 2009), tartaric (Koleva *et al.*, 2008) and squaric acid (Koleva *et al.*, 2007) have been reported in the literature. Herewith we present the crystal structure of the title compound (I).

In (I) (Fig 1), the asymmetric unit consists of a 3,4-diaminopyridinium cation and a hydrogen malonate anion. In the 3,4-diaminopyridinium cation, endocyclic angles cover the range 117.98 (18)–121.95 (18)°. Protonation at atom N1 has led to slight increase in the C2—N1—C6 angle to 121.32 (2)° compared to that in unprotonated structure (De Cires-Mejias *et al.*, 2004). All non-hydrogen atoms lie within the same plane (r.m.s. deviation is 0.02 Å). The dihedral angle between the pyridine ring and the plane formed by the malonic acid molecule is 5.08 (6) °. Hydrogen malonate anion is stabilized by intramolecular O4—H···O2 hydrogen bond.

In the crystal, the protonated N1 atom is bonded to the carboxylate oxygen atom O3 through N—H···O hydrogen bond. The two amino groups (N2 and N3) are involved in the hydrogen bonding *via* N—H···O H-bonds with hydrogen malonate oxygen atom (O1) to form an *R*^1^_2_(7) ring motif (Bernstein *et al.*, 1995). The N3 amino group is hydrogen-bonded to the carboxylate oxygen atom (O3). The N—H···O hydrogen bonds (Table 1) link cations and anions into layers parallel to *ab* plane (Fig. 2).

### Experimental

The initial compounds were obtained commercially (Aldrich) as fine-crystalline powders and purified additionally by filtration. 0.003 g (0.028 mmol) of malonic acid and 0.0035 (0.032 mmol) of 3,4 diaminopyridine were disolved in hot ethanol. Crystals suitable for the X-ray diffraction study were obtained after couple of days by slow evaporation (m.p.:130–135°C).

### Refinement

The hydrogen atom of hydroxyl group was localized in the difference-Fourier map and refined isotropically. The other hydrogen atoms were placed in the calculated positions with N—H = 0.86 Å, C—H = 0.93 Å (aromatic) and C—H = 0.97 Å (methylene) and refined in the riding model with fixed isotropic displacement parameters [*U*_iso_(H) = 1.2*U*_eq_(N), *U*_iso_(H) = 1.2*U*_eq_(C)]. In the absence of any significant anomalous scatterers, the 369 Friedel pairs were merged before the final refinement.

### Crystal data

**Table:**

C_5_H_8_N_3_^+^·C_3_H_3_O_4_^−^	*F*(000) = 224
*M**_r_* = 213.20	*D*_x_ = 1.521 Mg m^−^^3^
Monoclinic, *P*2_1_	Mo *K*α radiation, λ = 0.71073 Å
Hall symbol: P 2yb	Cell parameters from 1070 reflections
*a* = 8.7761 (18) Å	θ = 2.8–27.0°
*b* = 5.088 (1) Å	µ = 0.12 mm^−^^1^
*c* = 10.636 (2) Å	*T* = 296 K
β = 101.381 (4)°	Prism, colourless
*V* = 465.58 (17) Å^3^	0.30 × 0.20 × 0.20 mm
*Z* = 2	

### Data collection

**Table:**

Bruker APEXII CCD diffractometer	1248 independent reflections
Radiation source: fine-focus sealed tube	1066 reflections with *I* > 2σ(*I*)
Graphite monochromator	*R*_int_ = 0.026
φ and ω scans	θ_max_ = 28.0°, θ_min_ = 2.0°
Absorption correction: multi-scan (*SADABS*; Sheldrick, 2003)	*h* = −11→11
*T*_min_ = 0.964, *T*_max_ = 0.976	*k* = −6→6
3498 measured reflections	*l* = −14→14

### Refinement

**Table:**

Refinement on *F*^2^	Primary atom site location: structure-invariant direct methods
Least-squares matrix: full	Secondary atom site location: difference Fourier map
*R*[*F*^2^ > 2σ(*F*^2^)] = 0.040	Hydrogen site location: inferred from neighbouring sites
*wR*(*F*^2^) = 0.102	H atoms treated by a mixture of independent and constrained refinement
*S* = 1.18	*w* = 1/[σ^2^(*F*_o_^2^) + (0.0456*P*)^2^ + 0.0455*P*] where *P* = (*F*_o_^2^ + 2*F*_c_^2^)/3
1248 reflections	(Δ/σ)_max_ < 0.001
140 parameters	Δρ_max_ = 0.19 e Å^−^^3^
1 restraint	Δρ_min_ = −0.15 e Å^−^^3^

### Special details

**Table:**

Geometry. All e.s.d.'s (except the e.s.d. in the dihedral angle between two l.s. planes) are estimated using the full covariance matrix. The cell e.s.d.'s are taken into account individually in the estimation of e.s.d.'s in distances, angles and torsion angles; correlations between e.s.d.'s in cell parameters are only used when they are defined by crystal symmetry. An approximate (isotropic) treatment of cell e.s.d.'s is used for estimating e.s.d.'s involving l.s. planes.
Refinement. Refinement of *F*^2^ against ALL reflections. The weighted *R*-factor *wR* and goodness of fit *S* are based on *F*^2^, conventional *R*-factors *R* are based on *F*, with *F* set to zero for negative *F*^2^. The threshold expression of *F*^2^ > σ(*F*^2^) is used only for calculating *R*-factors(gt) *etc*. and is not relevant to the choice of reflections for refinement. *R*-factors based on *F*^2^ are statistically about twice as large as those based on *F*, and *R*- factors based on ALL data will be even larger.

### Fractional atomic coordinates and isotropic or equivalent isotropic displacement parameters (Å^2^)

**Table:**

	*x*	*y*	*z*	*U*_iso_*/*U*_eq_	
N1	0.3630 (3)	0.6491 (5)	0.2325 (2)	0.0467 (6)	
H1A	0.4198	0.5329	0.2052	0.056*	
N2	0.0891 (3)	1.1609 (5)	0.09714 (19)	0.0461 (6)	
H2A	0.0922	1.1490	0.0170	0.055*	
H2B	0.0283	1.2730	0.1225	0.055*	
N3	0.0818 (3)	1.1851 (5)	0.3616 (2)	0.0428 (5)	
H3A	0.0792	1.1894	0.4420	0.051*	
H3B	0.0253	1.2917	0.3094	0.051*	
C2	0.2794 (3)	0.8205 (5)	0.1477 (3)	0.0422 (6)	
H2	0.2893	0.8147	0.0623	0.051*	
C3	0.1811 (3)	1.0013 (5)	0.1844 (2)	0.0333 (5)	
C4	0.1737 (3)	1.0129 (5)	0.3178 (2)	0.0335 (5)	
C5	0.2662 (3)	0.8383 (6)	0.4014 (3)	0.0423 (6)	
H5	0.2649	0.8450	0.4886	0.051*	
C6	0.3587 (3)	0.6579 (6)	0.3574 (3)	0.0485 (7)	
H6	0.4184	0.5416	0.4142	0.058*	
O1	0.8907 (2)	−0.4299 (4)	0.19036 (19)	0.0501 (5)	
O2	0.7898 (2)	−0.2740 (5)	0.35111 (17)	0.0536 (6)	
O3	0.5470 (2)	0.2937 (4)	0.13031 (19)	0.0483 (5)	
O4	0.6154 (3)	0.1047 (4)	0.32045 (18)	0.0494 (5)	
H4	0.674 (4)	−0.042 (7)	0.345 (3)	0.044 (9)*	
C7	0.8059 (3)	−0.2775 (5)	0.2335 (2)	0.0356 (6)	
C8	0.7139 (3)	−0.0761 (5)	0.1443 (2)	0.0340 (5)	
H8A	0.7865	0.0192	0.1034	0.041*	
H8B	0.6444	−0.1711	0.0774	0.041*	
C9	0.6182 (3)	0.1235 (5)	0.1993 (2)	0.0348 (5)	

### Atomic displacement parameters (Å^2^)

**Table:**

	*U*^11^	*U*^22^	*U*^33^	*U*^12^	*U*^13^	*U*^23^
N1	0.0438 (12)	0.0339 (12)	0.0626 (14)	0.0032 (11)	0.0108 (10)	−0.0094 (12)
N2	0.0563 (13)	0.0490 (14)	0.0309 (10)	0.0087 (12)	0.0035 (9)	0.0022 (10)
N3	0.0515 (12)	0.0438 (13)	0.0325 (10)	0.0068 (11)	0.0071 (9)	−0.0015 (10)
C2	0.0424 (14)	0.0394 (15)	0.0431 (13)	−0.0060 (13)	0.0043 (11)	−0.0061 (12)
C3	0.0334 (12)	0.0334 (13)	0.0324 (11)	−0.0062 (11)	0.0043 (9)	−0.0017 (10)
C4	0.0359 (12)	0.0296 (12)	0.0337 (11)	−0.0025 (11)	0.0039 (9)	−0.0014 (10)
C5	0.0464 (14)	0.0396 (15)	0.0374 (12)	0.0013 (13)	0.0002 (11)	0.0068 (11)
C6	0.0493 (16)	0.0365 (14)	0.0565 (16)	0.0019 (13)	0.0027 (12)	0.0082 (14)
O1	0.0502 (11)	0.0430 (12)	0.0555 (11)	0.0150 (10)	0.0066 (9)	0.0097 (10)
O2	0.0702 (14)	0.0551 (13)	0.0323 (9)	−0.0030 (11)	0.0025 (8)	0.0095 (9)
O3	0.0470 (11)	0.0402 (11)	0.0558 (11)	0.0132 (9)	0.0055 (8)	−0.0025 (9)
O4	0.0606 (12)	0.0508 (13)	0.0380 (10)	0.0003 (12)	0.0129 (9)	−0.0113 (10)
C7	0.0361 (13)	0.0345 (13)	0.0340 (12)	−0.0029 (11)	0.0014 (9)	0.0048 (11)
C8	0.0381 (12)	0.0363 (13)	0.0268 (10)	0.0051 (11)	0.0044 (9)	0.0028 (10)
C9	0.0328 (12)	0.0346 (13)	0.0363 (11)	−0.0054 (11)	0.0053 (9)	−0.0038 (11)

### Geometric parameters (Å, º)

**Table:**

N1—C6	1.337 (4)	C5—C6	1.368 (4)
N1—C2	1.360 (4)	C5—H5	0.9300
N1—H1A	0.8600	C6—H6	0.9300
N2—C3	1.370 (3)	O1—C7	1.225 (3)
N2—H2A	0.8600	O2—C7	1.286 (3)
N2—H2B	0.8600	O3—C9	1.224 (3)
N3—C4	1.335 (3)	O4—C9	1.298 (3)
N3—H3A	0.8600	O4—H4	0.91 (3)
N3—H3B	0.8600	C7—C8	1.517 (3)
C2—C3	1.369 (4)	C8—C9	1.507 (3)
C2—H2	0.9300	C8—H8A	0.9700
C3—C4	1.435 (3)	C8—H8B	0.9700
C4—C5	1.398 (3)		
			
C6—N1—C2	121.3 (3)	C6—C5—H5	119.4
C6—N1—H1A	119.4	C4—C5—H5	119.4
C2—N1—H1A	119.4	N1—C6—C5	119.9 (3)
C3—N2—H2A	120.0	N1—C6—H6	120.1
C3—N2—H2B	120.0	C5—C6—H6	120.1
H2A—N2—H2B	120.0	C9—O4—H4	102.8 (19)
C4—N3—H3A	120.0	O1—C7—O2	124.4 (2)
C4—N3—H3B	120.0	O1—C7—C8	118.7 (2)
H3A—N3—H3B	120.0	O2—C7—C8	116.9 (2)
N1—C2—C3	122.0 (2)	C9—C8—C7	118.75 (19)
N1—C2—H2	119.0	C9—C8—H8A	107.6
C3—C2—H2	119.0	C7—C8—H8A	107.6
C2—C3—N2	121.7 (2)	C9—C8—H8B	107.6
C2—C3—C4	117.6 (2)	C7—C8—H8B	107.6
N2—C3—C4	120.7 (2)	H8A—C8—H8B	107.1
N3—C4—C5	120.7 (2)	O3—C9—O4	122.7 (2)
N3—C4—C3	121.4 (2)	O3—C9—C8	120.1 (2)
C5—C4—C3	118.0 (2)	O4—C9—C8	117.2 (2)
C6—C5—C4	121.2 (2)		
			
C6—N1—C2—C3	−3.3 (4)	C3—C4—C5—C6	−1.4 (4)
N1—C2—C3—N2	−175.4 (3)	C2—N1—C6—C5	1.4 (4)
N1—C2—C3—C4	2.7 (4)	C4—C5—C6—N1	0.9 (4)
C2—C3—C4—N3	179.4 (3)	O1—C7—C8—C9	−175.8 (2)
N2—C3—C4—N3	−2.5 (4)	O2—C7—C8—C9	3.6 (4)
C2—C3—C4—C5	−0.4 (3)	C7—C8—C9—O3	177.5 (2)
N2—C3—C4—C5	177.7 (2)	C7—C8—C9—O4	−2.7 (3)
N3—C4—C5—C6	178.8 (3)		

### Hydrogen-bond geometry (Å, º)

**Table:**

*D*—H···*A*	*D*—H	H···*A*	*D*···*A*	*D*—H···*A*
N1—H1*A*···O3	0.86	1.93	2.784 (3)	175
N2—H2*A*···O1^i^	0.86	2.28	3.132 (3)	174
N2—H2*B*···O1^ii^	0.86	2.15	3.005 (3)	176
N3—H3*A*···O2^iii^	0.86	2.28	3.048 (3)	149
N3—H3*B*···O1^ii^	0.86	2.10	2.960 (3)	177
O4—H4···O2	0.91 (3)	1.55 (3)	2.442 (3)	164 (3)

Symmetry codes: (i) −*x*+1, *y*+3/2, −*z*; (ii) *x*−1, *y*+2, *z*; (iii) −*x*+1, *y*+3/2, −*z*+1.
